# Isobaric Vapor–Liquid Equilibrium Data for Tetrahydrofuran + Acetic Acid and Tetrahydrofuran + Trichloroethylene Mixtures

**DOI:** 10.1021/acs.jced.2c00593

**Published:** 2023-01-27

**Authors:** Vyomesh M. Parsana, Sachin Parikh, Keval Ziniya, Hirvita Dave, Piyush Gadhiya, Kedar Joshi, Dolly Gandhi, Thijs J. H. Vlugt, Mahinder Ramdin

**Affiliations:** † Chemical Engineering Department, V.V.P. Engineering College, Gujarat Technological University, Rajkot-360005, Gujarat, India; ‡ Department of Chemical Engineering, L.D. College of Engineering, Gujarat Technological University, Ahmedabad-380015, Gujarat, India; § Department of Chemical Engineering, Government Polytechnic Rajkot, Rajkot-360003, Gujarat, India; ∥ Chemical Engineering Department, Vishwakarma Government Engineering College, Gujarat Technological University, Ahmedabad-382424, Gujarat, India; ⊥ Engineering Thermodynamics, Process & Energy Department, Faculty of Mechanical, Maritime and Materials Engineering, Delft University of Technology, Leeghwaterstraat 39, 2628 CBDelft, The Netherlands

## Abstract

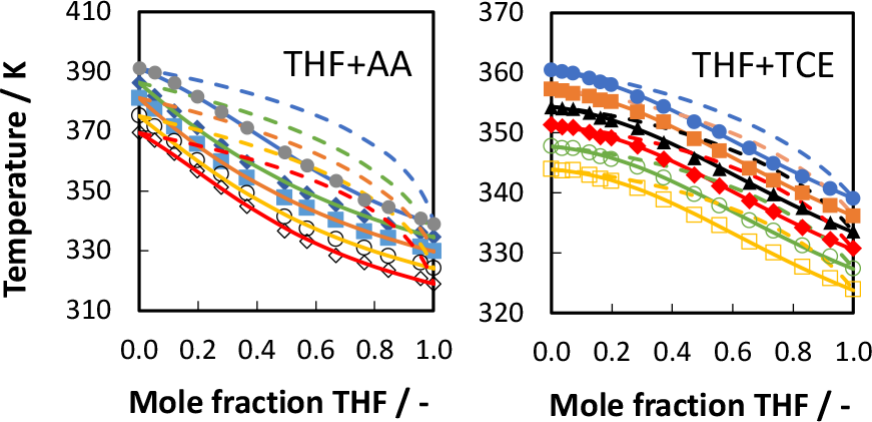

Vapor–liquid equilibrium (VLE) data for the binary systems tetrahydrofuran (THF) + acetic acid (AA) and THF + trichloroethylene (TCE) were measured under isobaric conditions using an ebulliometer. The boiling temperatures for the systems (THF + AA/THF + TCE) are reported for 13/15 compositions and five/six different pressures ranging from 50.2/60.0 to 101.1/101.3 kPa, respectively. The THF + AA system shows simple phase behavior with no azeotrope formation. The THF + TCE system does not exhibit azeotrope formation but seems to have a pinch point close to the pure end of TCE. The nonrandom two-liquid (NRTL) and universal quasichemical (UNIQUAC) activity coefficient models were used to accurately fit the binary (*PTx*) data. Both models were able to fit the binary VLE data satisfactorily. However, the NRTL model was found to be slightly better than UNIQUAC model in fitting the VLE data for both systems. The results can be used for designing liquid–liquid extraction and distillation processes involving mixtures of THF, AA, and TCE.

## Introduction

Acetic acid (AA) is an important base chemical that is used in a variety of applications. The majority of acetic acid produced worldwide is used for vinyl acetate (polymers), acetic anhydride, acetate esters (cellulose), terephthalic acid, monochloroacetic acid, and others.^1^ In industry, AA is mainly produced from the carbonylation of methanol (Monsanto process), which is an energy-intensive process based on fossil fuels. Therefore, more sustainable alternatives for AA production, for example based on biomass hydrolysis, are recently being explored. Tetrahydrofuran (THF) is often used as a cosolvent in the lignocellulosic biomass hydrolysis process, which yields acetic acid as one of the main products.^2,3^ Therefore, the hydrolysate contains THF, acetic acid, and other byproducts. The cosolvent (THF) needs to be separated from the product mixture, which mainly contains tetrahydrofuran and acetic acid, and recycled back to the process.

Tetrahydrofuran, which is being explored as a green solvent for various processes, can be synthesized via dehydration of 1,4-butanediol. A mineral catalyst can be used in the synthesis, but it gets neutralized after the reaction and generates salt waste. A more eco-friendly approach is to use high-temperature liquid water as the reaction medium, where water also acts as the proton donor and eliminates necessity for a catalyst.^4^ Separation of tetrahydrofuran from its aqueous solution is very critical, as it forms azeotrope at 101.325 kPa and 63.43 °C. Thus, conventional distillation cannot be an effective technique to separate tetrahydrofuran and water.^5^ For this reason, often a solvent is used to extract tetrahydrofuran from the aqueous phase. Trichloroethylene (TCE) is an effective solvent for tetrahydrofuran extraction from aqueous solutions.^6^

To design such a separation process (THF + AA and THF + TCE), vapor–liquid equilibrium (VLE) data for these systems are required. The vapor–liquid equilibrium of tetrahydrofuran and acetic acid was studied at 303.15 and 323.15 K (isothermal conditions) by Kalali et al.,^7^ but isobaric VLE data are lacking for this system. We note that isobaric data are more practical for distillation processes, since the distillation column is typically operated at constant pressure rather than at constant temperature. Furthermore, the temperature range studied by Kalali et al.^7^ is far from the boiling points of both components at atmospheric pressure. Isobaric vapor–liquid equilibrium data for tetrahydrofuran + acetic acid were predicted by Ziniya et al.^8^ using the UNIFAC method, and those for tetrahydrofuran + tetrachloroethylene were predicted by Joshi et al.^9^ using the UNIFAC and modified UNIFAC (Dortmund) methods at atmospheric pressure. To the best of our knowledge, experimental VLE data under isobaric conditions for the THF + AA system have not been reported before, while Prasad et al.^10^ reported the bubble points of the THF + TCE system at 95.8 kPa. We performed a reliability check of the VLE data for the THF + TCE system reported by Prasad et al.^10^ and found some inconsistencies in their vapor pressure data. The details of this reliability check of the VLE data are presented in the Results and Discussion. The main objective of this study is to generate experimental vapor–liquid equilibrium data which are essential to design separation system for the THF + AA and THF + TCE binary systems. For binary systems, two kind of measurement approaches are available: (1) *PTxy* measurements using a dynamic VLE still and (2) *PTx* measurements using an ebulliometer. The measurement of *y* (vapor phase composition) is not reliable and increases the uncertainty in VLE data.^11^ The *PTxy* measurement approach is time-consuming and requires large amounts of chemicals. It does not add much to VLE data;^11^ however, it allows Gibbs–Duhem verification. Parsana and Parikh adopted this method in the measurement of isobaric VLE data for the 2-methyltetrahydrofuran + acetic acid system at 101.3 kPa.^12^

The second approach, *PTx* measurement, overcomes the above-mentioned limitations. For VLE data measured under vacuum, the *PTx* approach is better, as we can assume that the vapor phase is ideal. Accurate VLE data for the binary systems 2-methyltetrahydrofuran + formic acid,^13^ 2-ethoxyethanol + toluene, and 2-ethoxyethyl acetate + toluene have been measured at atmospheric pressure and vacuum pressure.^14^ An ebulliometer was used to measure the boiling points of THF + AA and THF + TCE mixtures at five and six different pressures, respectively. The nonrandom two liquid (NRTL) and universal quasichemical (UNIQUAC) activity coefficient models were used to accurately correlate the experimental data.

This article is organized as follows. In the Experimental Section, we provide the details of the experimental VLE measurements. In Thermodynamic Modeling, we present the details of the VLE calculations. In the Results and Discussion, the experimental and modeling results are discussed. In the Conclusions, the main findings of our work are summarized.

## Experimental Section

The chemicals used for the experimentation were of analytical grade and purchased from LOBA Chemie Pvt. Ltd., Mumbai. The purities of chemicals reported by the supplier were verified by measurements of the refractive indices of the pure compounds. These chemicals were used without further purification. Detailed information on the chemicals is provided in Table 1.

**Table 1:** Purities and Supplier of Chemicals^a^

chemical	CAS number	supplier	purity (%)	*n*_D_(293.15 K)^b^
acetic acid (AA)	64-19-7	LOBA Chemie Pvt. Ltd.	99.7	1.3692^c^/1.3717^24^
tetrahydrofuran (THF)	109-99-9	LOBA Chemie Pvt. Ltd.	>99.0	1.4062^c^/1.4073^25^
trichloroethylene (TCE)	79-01-6	LOBA Chemie Pvt. Ltd.	>99.5	1.4763^d^/1.4773^26^

a Standard uncertainties: *u*(*n*_D_) = 0.0003, *u*(*P*) = 0.133 kPa, *u*(*T*) = 0.5 K.

b Experimental value from this work/literature value.

c *n*_D_ was measured at 101.1 kPa,

d *n*_D_ was measured at 101.3 kPa,

A schematic diagram of the experimental setup is shown in Figure 1. A modified ebulliometer was used to measure the boiling points of THF + AA and THF + TCE mixtures at five and six different pressures ranging from 50.2 to 101.1 kPa and 60.0 to 101.3 kPa, respectively. Thirteen and 15 mixtures of the THF + AA and THF + TCE systems, respectively, covering the whole composition range were studied. The pressure was measured using a mercury-filled U-tube manometer with a precision of 0.133 kPa (1 mmHg). The equilibrium temperature was measured with a calibrated Pt-100 temperature sensor with a precision of 0.1 K. Before the experiments were run, the setup was thoroughly cleaned with acetone to remove possible impurities in the ebulliometer and condenser. The setup was kept under vacuum for a few hours to remove any traces of residual chemicals to avoid contamination of samples. The setup was validated by checking the boiling points of the pure components. In Table 2, a comparison of the measured vapor pressures with vapor pressures from the literature calculated with the Antoine parameters (reported in Table 3) is provided. In Figure 2, a plot of the residuals of the vapor pressure versus boiling temperature is shown. The comparison shows that the variation in the vapor pressures is less than 0.5% (except for one data point with a deviation of 0.97%). Hence, the modified ebulliometer is reliable for the experimental measurement of the boiling temperatures of the binary systems under study. The experiments were started by charging an approximately 60 mL sample into the ebulliometer. The sample mixture in the heating chamber was heated by a belt heater. The heating rate was increased very slowly to prevent bumping and superheating of the liquid inside the equilibrium chamber. The heating rate was controlled precisely by a voltage regulator. The cooling water flow was started before the initiation of the heater to avoid any loss of material. The temperature and the drop rate were monitored closely to determine the equilibrium state. The system was considered to be in equilibrium once the temperature and drop rate were constant. The time taken to reach the equilibrium state was approximately 30 to 40 min. The modified ebulliometer was equipped with a vacuum pump to maintain a lower pressure in the system. The ballast tank was provided to minimize the fluctuations in the pressure while lowering the pressure to the desired level through a manually operated valve. The first set of data was obtained at atmospheric pressure. In the next experiment, the pressure was changed by applying a vacuum and adjusting the heating rate accordingly. *PTx* diagrams were generated by repeating this procedure for different pressures and compositions. It is important to note that the gas-phase composition cannot be obtained by ebulliometry. The composition of the liquid samples at the end of the experiments was analyzed with a five-digit automatic digital refractometer (RFM-950 supplied by LABMAN). Its measurement range is 1.30000–1.70000. The measuring accuracy of the instrument is ±0.00002 as stated by the supplier. The refractometer was cleaned properly before and after analysis of each sample. The calibration curve was prepared by plotting refractive index values of different samples of known composition of THF + AA and THF + TCE systems with *R*^2^ = 0.998 and 0.999, respectively. The uncertainty in the reported composition is 0.001 in the mole fraction.

**Figure 1:**
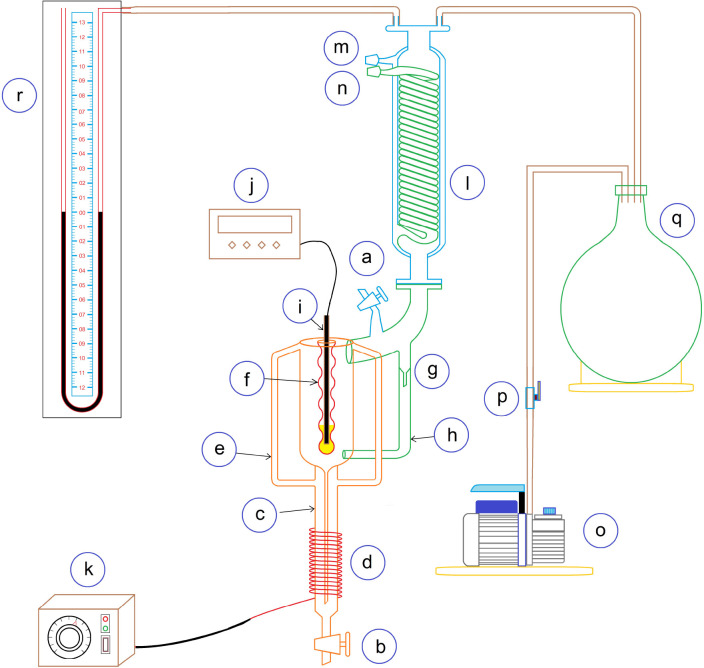
Experimental setup used for the vapor–liquid equilibrium measurements: (a) feed point; (b) drain valve; (c) heating chamber; (d) belt heater; (e) Cottrell tube; (f) thermowell; (g) drop counter; (h) liquid return line; (i) Pt-100 temperature sensor; (j) temperature display; (k) voltage regulator; (l) jacketed internal coil condenser; (m) cooling water outlet; (n) cooling water inlet; (o) vacuum pump; (p) pressure regulator; (q) glass ballast tank; (r) U-tube mercury manometer.

**Figure 2:**
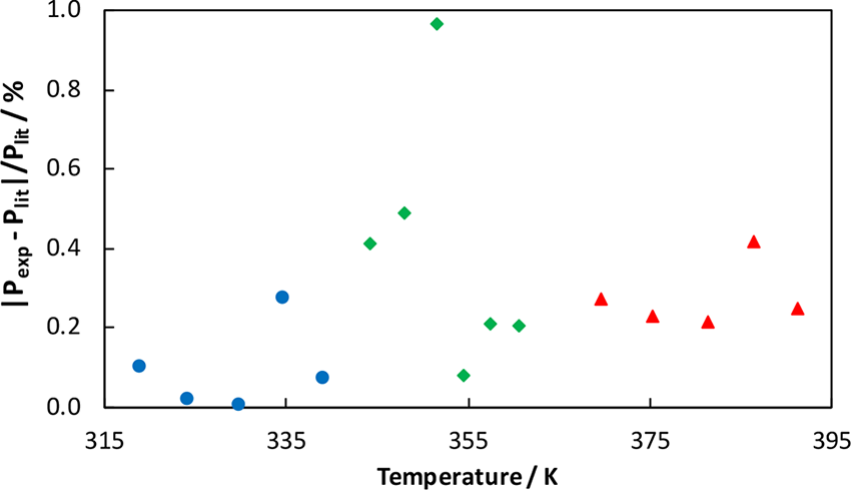
Residual plot of vapor pressure vs temperature for AA (triangles), THF (circles), and TCE (diamonds).

**Table 2:** Comparison of Measured Vapor Pressures with Literature Vapor Pressure Data for AA, THF, and TCE

*T*/K	*P*_exp_/kPa	*P*_lit_/kPa^a^	/%
AA			
391.1	101.1	101.4	0.25
386.3	87.0	87.4	0.42
381.2	74.1	74.3	0.22
375.2	60.8	60.9	0.23
369.6	50.2	50.3	0.27
THF			
339.1	101.1	101.2	0.07
334.6	87.0	87.2	0.27
329.8	74.1	74.1	0.00
324.2	60.8	60.8	0.02
319.0	50.2	50.2	0.10
TCE			
360.6	101.3	101.5	0.20
357.4	92.0	92.2	0.21
354.4	84.0	84.1	0.08
351.5	76.0	76.7	0.97
347.9	68.0	68.3	0.49
344.1	60.0	60.2	0.41

a Literature vapor pressure data from Poling et al.^16^ for AA and THF and from NIST^17^ for TCE.

**Table 3:** Antoine Parameters for Acetic Acid, Tetrahydrofuran, and Trichloroethylene^a^

component	*A*	*B*	*C*	*D*	normal boiling point/K^b^	ref
AA	4.545	1555.120	224.650	273.15	391.1/391.0	(16)
THF	4.121	1203.110	226.355	273.15	339.1/339.1	(16)
TCE	3.553	974.540	–85.811	0	360.6/360.2	(17)

a log_10_(*P*_*i*_^sat^/bar) = *A* – {*B*/[*C* + (*T*/K) – *D*]}.

b Experimental value of this work/literature value.

## Thermodynamic Modeling

The experimental VLE data for both systems were modeled with the γ–ϕ approach, which can be written as^15^
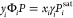
1where *y*_*i*_ is the gas-phase composition, Φ_*i*_ = (ϕ̂_*i*_/ϕ_*i*_^sat^) exp(−*V*_*i*_^l^(*P* – *P*_*i*_^sat^)/*RT*), *x*_*i*_ is the liquid-phase composition, γ_*i*_ is the activity coefficient, *P*_*i*_^sat^ is the saturation pressure, *P* is the total pressure, and *i* is the component number. In eq 1, Φ_*i*_ is assumed to be 1, which is a reasonable assumption for low pressures. The saturated vapor pressures of the components were measured and correlated with an Antoine-type equation:
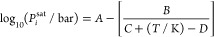
2The constants *A*, *B*, and *C* for AA, THF, and TCE are provided in Table 3. *D* is either 0 or 273.15 depending on the used reference. In ref (16), *D* is taken as 273.15, while ref (17) uses *D* = 0.

The experimental *PTx* data were correlated using the excess Gibbs energy function and activity coefficients of binary systems in vapor–liquid equilibrium. The NRTL and UNIQUAC excess Gibbs energy models were used to determine the activity coefficients.^18,19^ The mathematical expression of the UNIQUAC model is more complex than that of the NRTL model. However, it contains only two adjustable parameters in comparison to three adjustable parameters for the NRTL model. Both models can fit the experimental data of binary systems containing a wide range of compounds. The NRTL model contains a third parameter, α_12_, which is also known as the nonrandomness parameter. Its value typically varies from 0.2 to 0.5. The UNIQUAC model contains two component structural parameters, the surface area parameter *q* and the volume parameter *r*, which are reported in Table 4. A more detailed description can be found in ref (16). The experimental data were fitted with the above models, and two binary interaction parameters (BIPs) were obtained by the process called data reduction for both local-composition models, NRTL and UNIQUAC. The fitting of isothermal VLE data is straightforward, but fitting of isobaric data requires more effort. The procedure for calculating the BIPs is as follows: The experimental *PTx* data are considered as input. The total pressure *P*_cal_ is calculated as *P*_cal_ = ∑_*i*_ *x*_*i*_γ_*i*_*P*_*i*_^sat^, where *P*_*i*_^sat^ is calculated using the Antoine equation. The BIPs are obtained by minimizing the error between the experimental and calculated pressures.

**Table 4:** Values of the Volume Parameter *r* and Surface Area Parameter *q* for UNIQUAC Models^16^

compound	group(*k*)	main group no.	secondary group no.	*r*_*k*_	*q*_*k*_
THF	CH_2_	1	2	0.6744	0.540
	THF	13	27	0.9183	1.100
AA	CH_3_	1	1	0.9011	0.848
	COOH	20	42	1.3013	1.224
TCE	CH=C	2	8	0.8886	0.676
	Cl–(C=C)	37	69	0.7910	0.724

The error-minimizing function used here was the average absolute deviation (AAD) between the experimental pressure and the calculated pressure. The absolute deviation ensures that the errors with different signs do not cancel one another and do not show the overall error incorrectly. Equation 3 shows the objective function:
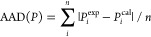
3where *n* is the number of data points and the BIPs are the input variables.

The procedure for calculating the vapor-phase mole fraction based on the NRTL or UNIQUAC model is as follows: The experimental *PTx* data and BIPs of the NRTL or UNIQUAC model are considered as input. Activity coefficients are calculated using the BIPs of the NRTL or UNIQUAC model. The experimental temperatures are taken as initial guesses, and at these temperatures vapor pressures are calculated using the Antoine equation. The total pressure *P*_cal_ is calculated as *P*_cal_ = ∑_*i*_ *x*_*i*_γ_*i*_*P*_*i*_^sat^, where *P*_*i*_^sat^ is calculated using Antoine constants. The error between the experimental pressure and the calculated pressure is minimized to obtain *T*_cal_ using the objective function given in eq 3, while the initial temperatures are taken as input variables. The vapor-phase mole fraction is calculated as *y*_*i*_ = *x*_*i*_γ_*i*_*P*_*i*_^sat^/*P*. AAD(*T*) is calculated using eq 4:
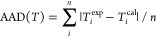
4

The Solver add-on (GRG Nonlinear solving method) in Microsoft Excel was used to perform the above calculations.

## Results and Discussion

The measured experimental vapor–liquid equilibrium data for the binary systems tetrahydrofuran + acetic acid and tetrahydrofuran + trichloroethylene are reported in Tables 5 and 6, respectively. The reported data are *P* (in kPa), *T* (in K), and *x*_1_ (mole fraction of tetrahydrofuran in the liquid phase).

**Table 5:** Experimental Isobaric Vapor–Liquid Equilibrium Data for the Binary System THF (1) + AA (2)^a^

101.1 kPa		87.0 kPa		74.1 kPa		60.8 kPa		50.2 kPa	
*x*_1_	*T*/K	*x*_1_	*T*/K	*x*_1_	*T*/K	*x*_1_	*T*/K	*x*_1_	*T*/K
0.000	391.1	0.000	386.3	0.000	381.2	0.000	375.2	0.000	369.6
0.052	389.8	0.052	381.8	0.052	377.4	0.052	371.7	0.052	367.1
0.119	386.3	0.119	376.8	0.119	371.6	0.119	366.7	0.119	362.7
0.196	381.6	0.196	371.3	0.196	365.7	0.196	360.3	0.196	356.8
0.278	376.5	0.278	365.4	0.278	359.9	0.278	355.7	0.278	351.3
0.366	371.2	0.366	359.7	0.366	354.4	0.366	349.3	0.366	345.0
0.492	363.1	0.492	352.7	0.492	347.8	0.492	341.5	0.492	336.7
0.569	358.6	0.569	349.3	0.569	344.3	0.569	337.4	0.569	333.3
0.668	353.5	0.668	345.5	0.668	340.3	0.668	334.0	0.668	328.5
0.764	347.2	0.764	341.7	0.764	336.5	0.764	331.1	0.764	326.1
0.846	344.8	0.846	338.9	0.846	334.3	0.846	328.3	0.846	323.5
0.954	340.9	0.954	336.1	0.954	331.3	0.954	326.1	0.954	320.7
1.000	339.1	1.000	334.6	1.000	329.8	1.000	324.2	1.000	319.0

a Standard uncertainties: *u*(*T*) = 0.5 K, *u*(*P*) = 0.133 kPa, *u*(*x*) = 0.001.

**Table 6:** Experimental Isobaric Vapor–Liquid Equilibrium Data for the Binary System THF (1) + TCE (2)^a^

101.3 kPa		92.0 kPa		84.0 kPa		76.0 kPa		68.0 kPa		60.0 kPa	
*x*_1_	*T*/K	*x*_1_	*T*/K	*x*_1_	*T*/K	*x*_1_	*T*/K	*x*_1_	*T*/K	*x*_1_	*T*/K
0.000	360.6	0.000	357.4	0.000	354.4	0.000	351.5	0.000	347.9	0.000	344.1
0.037	360.3	0.037	357.1	0.037	354.2	0.037	351.1	0.037	347.6	0.037	343.8
0.073	360.0	0.073	356.7	0.073	354.1	0.073	351.0	0.073	347.6	0.073	343.9
0.127	359.2	0.127	356.2	0.127	353.5	0.127	350.2	0.127	346.8	0.127	343.1
0.162	358.6	0.162	355.6	0.162	352.7	0.162	349.5	0.162	346.2	0.162	342.5
0.198	358.1	0.198	355.3	0.198	352.2	0.198	349.3	0.198	345.7	0.198	342.1
0.286	356.1	0.286	353.6	0.286	350.9	0.286	347.9	0.286	344.5	0.286	340.9
0.372	354.5	0.372	351.9	0.372	348.6	0.372	345.7	0.372	342.6	0.372	339.0
0.473	351.9	0.473	349.1	0.473	345.9	0.473	343.1	0.473	339.9	0.473	336.5
0.557	350.3	0.557	347.1	0.557	344.1	0.557	341.3	0.557	338.0	0.557	334.7
0.655	347.6	0.655	344.2	0.655	341.8	0.655	338.8	0.655	335.5	0.655	332.0
0.735	345.0	0.735	342.2	0.735	339.9	0.735	337.0	0.735	333.8	0.735	330.2
0.830	342.8	0.830	340.1	0.830	337.6	0.830	334.4	0.830	331.4	0.830	327.8
0.924	340.8	0.924	338.0	0.924	335.2	0.924	332.4	0.924	329.5	0.924	325.9
1.000	339.2	1.000	336.3	1.000	333.7	1.000	330.9	1.000	327.5	1.000	324.1

a Standard uncertainties: *u*(*T*) = 0.5 K, *u*(*P*) = 0.133 kPa, *u*(*x*) = 0.001.

**Figure 3:**
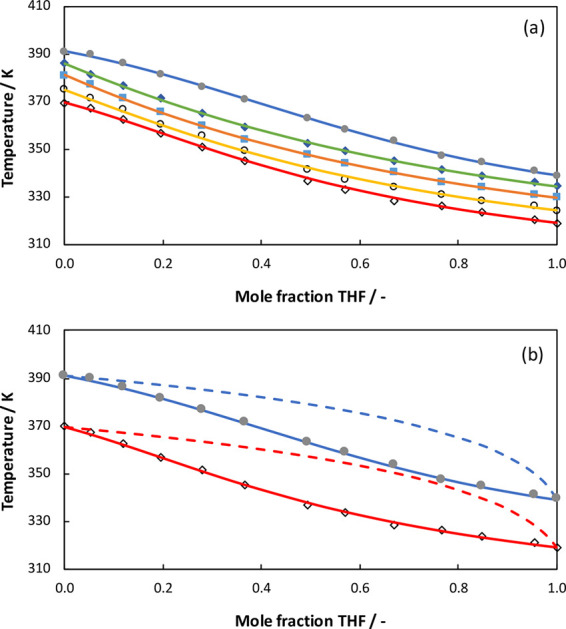
(a) Comparison of experimental data (symbols) and NRTL modeling results (solid lines) for the THF + AA system at different pressures: 101.1 kPa (closed circles), 87.0 kPa (closed diamonds), 74.1 kPa (squares), 60.8 kPa (open circles), and 50.2 kPa (open diamonds). (b) NRTL modeling results for the gas composition (dashed lines).

**Figure 4:**
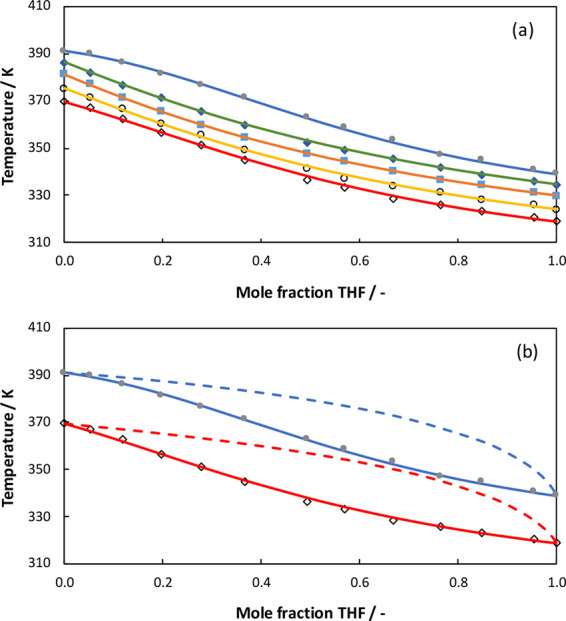
(a) Comparison of experimental data (symbols) and UNIQUAC modeling results (solid lines) for the THF + AA system at different pressures: 101.1 kPa (closed circles), 87.0 kPa (closed diamonds), 74.1 kPa (squares), 60.8 kPa (open circles), and 50.2 kPa (open diamonds). (b) UNIQUAC modeling results for the gas composition (dashed lines).

The experimental data and the NRTL modeling results for the THF + AA system are compared in Figure 3. In Figure 3a it can be seen that among the five pressure curves, the top curve representing the atmospheric pressure behaves differently than the other four pressure curves for mole fractions of THF between 0 and 0.5. A possible reason may include a phase behavior change from non-azeotropic to azeotropic at higher pressures, but this cannot be scrutinized here since our setup did not allow for measuring higher pressures and the vapor composition. Also, there is a tendency for acetic acid monomer to dimerize in the vapor phase at high pressure.^20^ It may not dimerize at low pressures, but the molecular behavior at low pressure cannot be predicted with the tools we have utilized in this work. We may scrutinize it in our subsequent work, or it can be explored further by other researchers. The optimized parameters for the NRTL model and the absolute average deviations (AADs) in the pressure and temperature can be found in Table 7. The ranges of AAD(*P*) and AAD(*T*) for the NRTL model are 0.47–0.94 and 0.20–0.46, respectively. The THF + AA system shows a simple phase behavior with no azeotrope formation at the studied pressures. The system shows a negative deviation from Raoult’s law. We can clearly see that the NRTL equation can accurately describe the phase behavior of the THF + AA system. In Figure 4, the experimental data and the UNIQUAC modeling results are presented. The fitted UNIQUAC parameters can be found in Table 8. The UNIQUAC model can also describe the experimental data well. The ranges of AAD(*P*) and AAD(*T*) for UNIQUAC model are 0.39–1.27 and 0.16–0.52, respectively. Though both the NRTL and UNIQUAC models could fit the experimental *PTx* data for THF + AA system quite well, the NRTL model provides a slightly better fit.

**Table 7:** Optimized NRTL Parameters at Different Pressures and Absolute Average Deviations in the Pressure and Temperature for the THF (1) + AA (2) and THF (1) + TCE (2) Systems

*P*/kPa	α_12_	Δ*g*_12_/(J/mol)	Δ*g*_21_/(J/mol)	AAD(*P*)	AAD(*T*)
THF (1) + AA (2)					
101.1	0.30	–543.88	–1543.69	0.94	0.30
87.0	0.30	–263.68	–258.37	0.54	0.20
74.1	0.30	–227.45	–218.26	0.47	0.20
60.8	0.47	–1693.72	1339.70	0.93	0.46
50.2	0.47	–2373.74	1801.16	0.74	0.43
THF (1) + TCE (2)					
101.3	0.30	–1206.54	63.03	0.40	0.13
92.0	0.30	–1430.90	196.80	0.29	0.10
84.0	0.30	–1120.90	–149.45	0.51	0.19
76.0	0.30	–1299.25	–36.58	0.47	0.19
68.0	0.30	–1398.89	67.88	0.41	0.18
60.0	0.30	–1430.08	63.39	0.37	0.18

**Table 8:** Optimized UNIQUAC Parameters at Different Pressures and Absolute Average Deviations in the Pressure and Temperature for the THF (1) + AA (2) and THF (1) + TCE (2) Systems

*P*/kPa	Δ*u*_12_/(J/mol)	Δ*u*_21_/(J/mol)	AAD(*P*)	AAD(*T*)
THF (1) + AA (2)				
101.1	–240.63	–640.32	1.27	0.42
87.0	87.45	–253.62	0.46	0.17
74.1	110.82	–244.78	0.39	0.16
60.8	57.41	–300.58	0.98	0.48
50.2	–64.58	–429.68	0.90	0.52
THF (1) + TCE (2)				
101.3	–90.00	–366.17	0.51	0.17
92.0	–828.17	401.11	0.29	0.10
84.0	–102.60	–404.18	0.58	0.22
76.0	–741.10	266.17	0.47	0.19
68.0	–606.33	117.44	0.41	0.18
60.0	–221.94	–317.96	0.32	0.16

**Figure 5:**
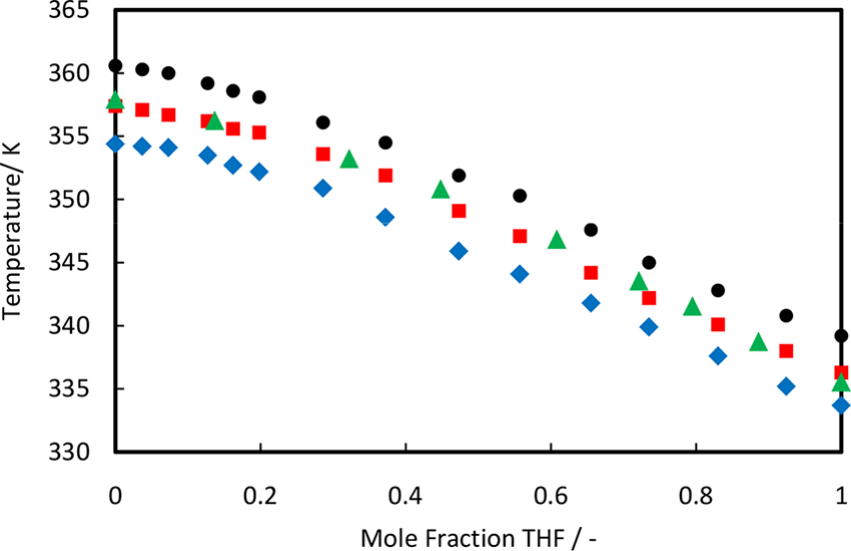
Comparison of experimental data for THF + TCE system: Prasad et al.^10^ at 95.8 kPa (triangles) and our work at 101.3 kPa (circles), 92.0 kPa (squares), and 84.0 kPa (diamonds) pressures.

**Figure 6:**
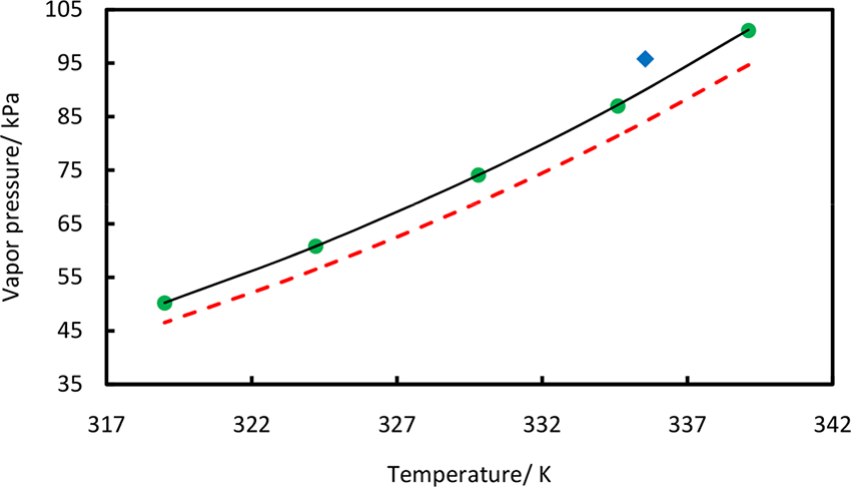
Comparison of vapor pressures of pure THF: Prasad et al.^10^ experimental point at 95.8 kPa (diamond), Antoine correlation of Prasad et al.^10^ (dashed line), our work (circles), and Poling et al.^16^ (solid line).

In Figure 5, a comparison is made between our experimental VLE data measured at 101.3, 92, and 84 kPa for THF + TCE system and the VLE data measured by Prasad et al.^10^ at 95.8 kPa for the same system. In Figure 6, we provide a comparison of the vapor pressure data for THF from Prasad et al.,^10^ our experiments, and the literature.^16^ These results shows that our experimental data coincide with literature data but the data of Prasad et al.^10^ deviate significantly from the literature data.^16^ Note that there is a discrepancy between their regressed data and their own experimental point.

**Figure 7:**
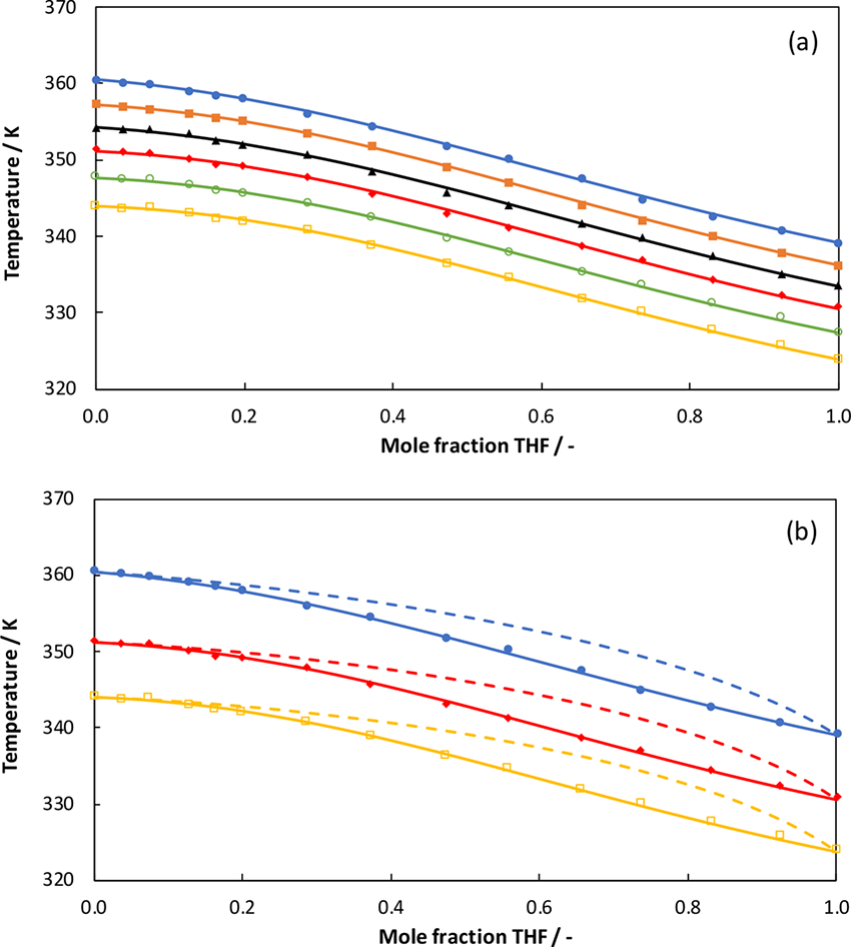
(a) Comparison of experimental data (symbols) and NRTL modeling results (solid lines) for the THF + TCE system at different pressures: 101.3 kPa (closed circles), 92.0 kPa (closed squares), 84.0 kPa (triangles), 76.0 kPa (closed diamonds), 68.0 kPa (open circles), and 60.0 kPa (open squares). (b) NRTL modeling results for the gas composition (dashed lines).

**Figure 8:**
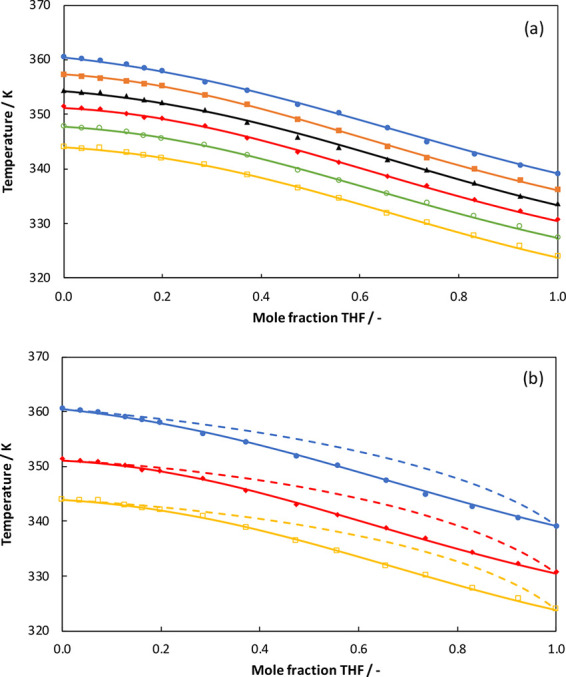
(a) Comparison of experimental data (symbols) and UNIQUAC modeling results (solid lines) for the THF + TCE system at different pressures: 101.3 kPa (closed circles), 92.0 kPa (closed squares), 84.0 kPa (triangles), 76.0 kPa (closed diamonds), 68.0 kPa (open circles), and 60.0 kPa (open squares). (b) UNIQUAC modeling results for the gas composition (dashed lines).

The experimental data and the NRTL modeling results for the THF + TCE system are compared in Figure 7. The UNIQUAC modeling results are compared in Figure 8. Figures 7a and 8a show *T*–*x* plots at different pressures for the THF (1) + TCE (2) system, and Figures 7b and 8b show *T*–*x* and *T*–*y* plots at three different pressures for the THF (1) + TCE (2) system. The ranges of AAD(*P*) and AAD(*T*) for the NRTL model are 0.29–0.51 and 0.10–0.19, respectively. The ranges of AAD(*P*) and AAD(*T*) for the UNIQUAC model are 0.29–0.58 and 0.10–0.22, respectively. Both models can describe the experimental data quite well, but the NRTL model has a slight edge over the UNIQUAC model in terms of fitting the experimental data for the THF + TCE system. The THF + TCE system does not show azeotrope formation but seems to have a pinch point close to the pure-TCE end. The pinch point region is almost independent of the pressure. This means that THF + TCE mixtures will be difficult to separate by normal distillation. Trichloroethylene is an economical solvent for liquid–liquid extraction of tetrahydrofuran from aqueous solutions considering the fact that most of the solvents have higher boiling temperatures than water,^6^ but the solvent recovery step will be complicated due to the presence of the pinch point. Therefore, it is better to find alternative solvents for THF extraction.

It is extremely challenging to verify the consistency of isobaric VLE (*PTx*) data. Gibbs–Duhem-type consistency tests are applicable only to *PTxy* data,^11^ but VLE measurements in an ebulliometer do not allow measurement of the gas composition.^21^ The consistency test of Olson^22^ based on the Gibbs–Helmholtz equation requires heat of mixing data, which are in general difficult to measure and not available for our systems. In the words of Wisniak,^23^ a correct thermodynamic consistency test for isobaric data requires measurements of the heat of mixing at the bubble point of a set of mixtures having different compositions, a task that is very hard if not impossible to carry out. Therefore, isobaric VLE (*PTx*) data should be verified by other means.

**Figure 9:**
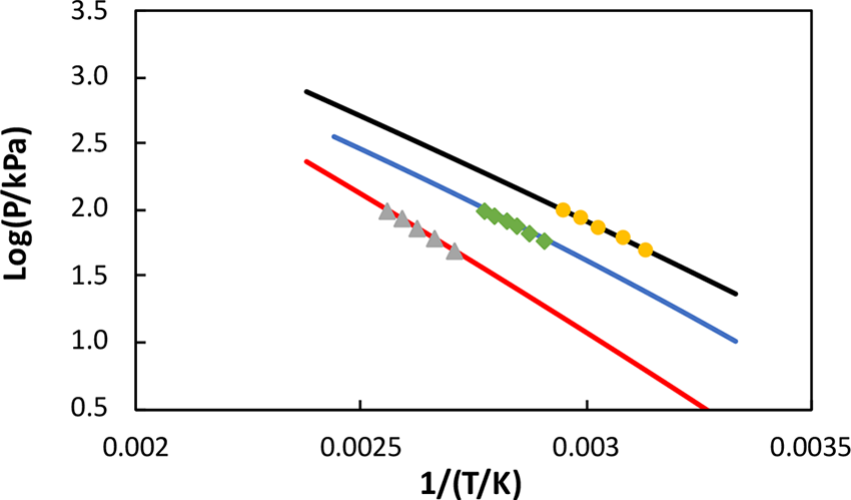
Comparison of pure-component vapor pressures from the Antoine equation (lines) and experiments (symbols) for AA (triangles), THF (circles), and TCE (diamonds).

**Figure 10:**
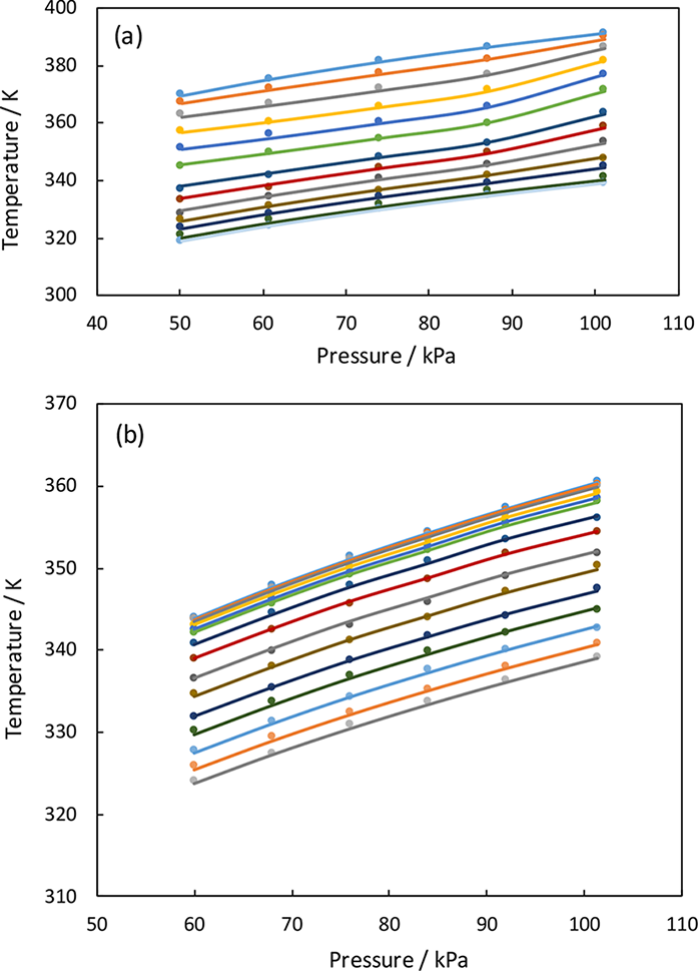
*P*–*T* diagrams at constant composition for the (a) THF + AA and (b) THF + TCE systems. Symbols are experimental data, and lines are NRTL modeling results.

We have verified our experimental results by checking the measured vapor pressures of the pure components with the Antoine equation, as shown in Figure 9. The deviations between the measured vapor pressures and vapor pressures calculated from the Antoine equation are plotted as residuals in Figure 2. The figure shows that a few points have error less than 0.1%, the majority of the points have error less than 0.5%, and only one point shows an error of 0.97%, which may be attributed to human error, etc. Overall, the measured pure-component vapor pressures are in good agreement with the Antoine equation. In Figure 10 we have plotted the experimental and computed *P*–*T* diagrams for the THF + AA and THF + TCE systems at constant composition. The *P*–*T* diagrams do not show any peculiarities (the boiling temperatures increase smoothly with pressure), which gives some confidence in the reliability of the measured data.

## Conclusions

A more sustainable alternative for acetic acid production requires a mixture of tetrahydrofuran and acetic acid to be separated. A more eco-friendly approach to tetrahydrofuran synthesis involves separation of tetrahydrofuran and water by extracting tetrahydrofuran from its aqueous mixture by an economical solvent like trichloroethylene, which is to be separated by distillation and recycled back. Thus, there is a requirement of VLE data for both the tetrahydrofuran + acetic acid and tetrahydrofuran + trichloroethylene systems. Due to unavailability of these data, we measured the VLE data for the THF + AA and THF + TCE systems at five and six different pressures, respectively, ranging from 50.2 to 101.3 kPa. The NRTL and UNIQUAC local-composition models were used to fit the experimental VLE data by regression.The average absolute deviation in pressure, AAD(*P*), and the average absolute deviation in temperature, AAD(*T*) were calculated for both binary systems. It has been found that the experimental VLE data were fitted slightly better with the NRTL activity coefficient model than the UNIQUAC model. However, both models could fit the VLE data accurately. The experimental and modeling findings show that THF + AA system shows a simple phase behavior with no azeotrope formation, while the THF + TCE system shows a pinch point close to the pure-TCE end. Consequently, normal distillation cannot be used to separate THF + TCE mixtures. Hence, it is a good idea to avoid TCE despite its economic benefit and to go for its alternative for the extraction of THF from its aqueous mixture. The results can be used to design liquid–liquid extraction and distillation systems involving mixtures of acetic acid, tetrahydrofuran, and trichloroethylene.
